# Evaluation of Primary Immunization Coverage of Infants Under Universal Immunization Programme in an Urban Area of Bangalore City Using Cluster Sampling and Lot Quality Assurance Sampling Techniques

**DOI:** 10.4103/0970-0218.42049

**Published:** 2008-07

**Authors:** Punith K, Lalitha K, Suman G, Pradeep BS, Jayanth Kumar K

**Affiliations:** 1 Department of Community Medicine, M.S. Ramaiah Medical College, Bangalore, Karnataka, India; 2 Department of Population Studies, St. Johns Research Institute, Bangalore, Karnataka, India

**Keywords:** Immunization coverage, cluster sampling and LQAS

## Abstract

**Research Question:**

Is LQAS technique better than cluster sampling technique in terms of resources to evaluate the immunization coverage in an urban area?

**Objective:**

To assess and compare the lot quality assurance sampling against cluster sampling in the evaluation of primary immunization coverage.

**Study Design:**

Population-based cross-sectional study.

**Study Setting:**

Areas under Mathikere Urban Health Center.

**Study Subjects:**

Children aged 12 months to 23 months.

**Sample Size:**

220 in cluster sampling, 76 in lot quality assurance sampling.

**Statistical Analysis:**

Percentages and Proportions, Chi square Test.

**Results:**

(1) Using cluster sampling, the percentage of completely immunized, partially immunized and unimmunized children were 84.09%, 14.09% and 1.82%, respectively. With lot quality assurance sampling, it was 92.11%, 6.58% and 1.31%, respectively. (2) Immunization coverage levels as evaluated by cluster sampling technique were not statistically different from the coverage value as obtained by lot quality assurance sampling techniques. Considering the time and resources required, it was found that lot quality assurance sampling is a better technique in evaluating the primary immunization coverage in urban area.

In 1985, the Universal Immunization Programme was started in India with the aim of achieving at least 85% coverage of primary immunization of infants, i.e. with three doses of DPT and OPV, one dose of BCG and one dose of measles by the year 1990.(1)

To evaluate the immunization coverage, the cluster sampling technique has been the most commonly used technique. But of late, lot quality sampling technique which was commonly used in the industrial set-up to assess the quality of the lots of their products is now used in the health services such as in evaluation of immunization coverage.

Since lot quality sampling method requires only a small sample size and easier for staff to use, it is feasible for routine monitoring of vaccination coverage.(2) In a study conducted in Rajasthan by NICD, New Delhi, the immunization coverage at PHC level estimated from lot quality assurance sampling (LQAS) was not significantly different from that estimated from cluster sampling technique.(3) Hence, an attempt was made to evaluate the primary immunization coverage in an urban area using LQAS versus cluster sampling technique.

## Objectives

- To assess the primary immunization coverage of infants in an urban area of Bangalore city.
- To compare lot quality assurance sampling against cluster sampling in the evaluation of primary immunization coverage in an urban area.

## Materials and Methods

The study was conducted in the areas under Mathikere Urban Health Center, field practice area of M.S. Ramaiah Medical College (population of 62,314) during August-September 2005. For the study, children of age between 12 and 23 months as on the date of survey were considered as the study subjects. The inclusion criteria For study subjects were those with availability of either an immunization card or a responsible person for key information regarding immunization and who were permanent residents (residing for more than 6 months) of the study area. Cluster sampling and LQAS were the sampling techniques employed for the evaluation. The coverage of BCG, OPV, DPT and measles vaccines was taken under consideration.

### Proof of Immunization

The child was considered as immunized or not, based on the immunization card. For those without an immunization card, information from the mother or any other responsible and reliable person in the family stating that the child has been immunized was considered. The lack of immunization cards was a common problem to both techniques but a shorter period of recall of child's immunization by the mother reduced this problem reduced the problem with that survey. If the mother could not remember regarding the vaccination or in the presence of any other confounding factors, the child was considered as not immunized with the vaccine under consideration. Child was considered as fully immunized if it received BCG (1), DPT (3), OPV (3) and measles (1); as Unimmunized if received none of these vaccines and Partially immunized if some dose given but immunization not complete.

### Calculation of sample size and sampling of unit

#### Cluster sampling

The sample size for the 30 by 7 cluster sample is based on a population proportion of 0.50, which yields the greatest estimate of variability, and therefore, the most conservative sample size. However, if the event of an immunized or unimmunized subject is very rare, a precision of ±10% would not be satisfactory. Hence, the sample size for cluster sampling was estimated based on the immunization coverage data of Bangalore urban for the year 2004 (data obtained from the district health office, Bangalore urban) and with a precision of 5%. The total sample size was calculated as 220. The calculation of the sample size for cluster sampling has been explained in Annexure 1.

The study area was first divided into 10 clusters based on the geographical demarcation and areas under link workers. The survey was carried out using the evaluation forms given in the Universal Immunization Programme (UIP) module(4) by trained personnel.

#### Lot quality assurance sampling (LQAS)

The sample size for LQAS is based on the hypothesized (or desired) immunization coverage. Keeping rarity of an event of getting an umimmunized child, the sample size was calculated.(5)

For LQAS, the sample size for each lot was taken as 19. This is the only lot size which has different decision rules for different coverage levels.(6) Four mutually exclusive subunits of the population were defined as a lot according to Auxiliary Nurse Midwife (ANM) areas (Supervisory areas). Therefore, total sample size for LQAS was 76. In each lot, the subunit was defined as area covered by a link worker.

The unit of study was the household having a child between 12 and 23 months. The acceptable level of immunization was defined as 85%. The standard set by UIP for primary immunization of infants. Hence, the decision rule was considered as 16. The decision rule serves as a benchmark for a lot to be considered acceptable or not acceptable. If the lot contains 16 or more immunized children for a particular antigen, then the coverage of immunization in that lot is acceptable. Otherwise, it is considered not acceptable. These 19 houses were sampled proportionately from different areas of the lot. The lot and sub-unit division and population proportionate sampling has been explained in Annexure 2.

The selection of house and proceeding with the survey was followed as mentioned for cluster sampling. The survey was carried out using the evaluation forms given in the UIP module.(4)

## Results and Discussions

The overall BCG coverage in cluster sampling was 97.72%, DPT and OPV coverage 92.27% each and measles coverage was 88.63% showing that the set goal of immunization was achieved [Table 1].

**Table 1:** Immunization coverage in various clusters* using cluster sampling technique

Cluster number	BCG	DPT(3)	Polio(3)	Measles
Cluster 1	20	18	18	15
Cluster 2	20	20	20	18
Cluster 3	21	19	19	17
Cluster 4	22	22	22	22
Cluster 5	22	21	21	22
Cluster 6	22	20	20	21
Cluster 7	22	22	22	22
Cluster 8	22	21	21	21
Cluster 9	22	19	20	18
Cluster 10	22	21	20	19
Grand total	215	203	203	195

* Each cluster contained 22 children

The overall BCG coverage in LQAS was 98.68%, DPT and OPV coverage 94.73% each and measles coverage was 92.11% during the study period. The coverage of the individual vaccines was above the 85% set goal of the UIP. The immunization coverage in all the lots was above the 85% acceptable level. Hence, all the lots were acceptable [Table 2].

**Table 2:** Immunization coverage in various LOTS^*^ using LQAS technique

LOT number	BCG	DPT(3)	Polio(3)	Measles
1	19	18	18	17
2	19	19	19	18
3	18	17	17	16
4	19	18	18	19
Grand total	75	72	72	70

* Each lot contained 19 children

The immunization status of the study area as per LQAS was above the acceptable level of immunization (i.e. above 85%) in all the Lots, whereas it was below acceptable level with cluster sampling in certain clusters but; however, the differences in the results of the two methodologies were not found to be statistically significant (*P* > 0.05) [Table 3].

**Table 3:** Immunization coverage of children aged 12-23 months in the study area using cluster sampling and LQAS techniques

Status	Cluster sampling no. (%)	LQAS no. (%)
Completely immunized	185 (84.09)	70 (92.11)
Partially immunized	31 (14.09)	5 (6.58)
Unimmunized	4 (1.82)	1 (1.31)
Total	220 (100)	76 (100)

The BCG coverage rate was found to be 98.63% in LQAS method, whereas it was 97.27% in cluster sampling methodologies. There was no significant difference in the vaccination coverage of other vaccines in both the methodologies [Table 4].

**Table 4:** Coverage level of different UIP vaccines by different techniques

Individual vaccines	Cluster sampling no. (%)	LQAS no. (%)
BCG	215 (97.72)	75 (98.63)
DPT	203 (92.27)	72 (94.73)
OPV	203 (92.27)	72 (94.73)
Measles	195 (88.63)	70 (92.11

Unaware of the need of immunization was the major reason for non-acceptance/discontinuation of immunization as obtained from both the methodologies. This is a problem of concern as knowing about the programme is just the first step towards an efficient immunization programme [Table 5].

**Table 5:** Top of mind reasons for failure of non-acceptance/discontinuation of immunization

Major reasons	Cluster sampling no. (%)	LQAS no. (%)
Unaware of need of immunization	21 (57.5)	3 (50.0)
Unaware of need to return for 2^nd^ or 3^rd^ dose	3 (8.57)	1 (16.67)
Lack of information about the place of immunization	1 (2.86)	0 (0.0)
Fear of side reaction	4 (11.42)	2 (33.34)
Postponed till another time	6 (17.14)	0 (0.0)
Total	35 (100)	6 (100)

Urban Health Center was the major source of immunization in the study area followed by private practitioners as seen in both the methodologies. Being an urban setup, private practitioners played a major role in the immunization, covering almost one-third of the population [Table 6].

**Table 6:** Percentage distribution of major sources of immunization in the study area

Major sources	Cluster sampling no. (%)*	LQAS no. (%)*
Health center	127 (58.79)	38 (50.67)
Private practitioners	58 (26.85)	26 (34.67)
Hospitals	25 (11.57)	10 (13.34)
Out-reach	6 (2.78)	1 (1.34)
Total*	216 (100)	75 (100)

* Includes completely and partially immunized subjects

Since the proportion of immunization coverage compared between cluster sampling and LQAS techniques was found to be statistically not significant [Table 3], application of the methodology requiring fewer resources for evaluating the vaccination coverage is economical. Hence, comparison of the resources required for the two methodologies to evaluate the vaccination coverage in our study is mentioned in Table 7.

**Table 7:** Comparison of resources required for cluster sampling and LQAS

Resources	Cluster sampling	LQAS
Number of people required for the evaluation	4	4
Number of divisions	10	4
Total number of households visited	3862	955
Average number of households visited per division	387	239
Total eligible children visited	220	76
Average number of households visited to obtain one eligible child	17	13
Man hours	224 man-hours	48 man-hours
Average time spent on survey per division	8 hours	6 hours
Total cost*	Rs. 3110	Rs. 1060

* Stationary, travel, printing, computer charges, etc

On comparing cluster sampling and LQAS methodologies with respect to man-hours, average time spent on survey per cluster/lot and total cost, the LQAS technique was found to be more economical as compared to cluster sampling technique for the evaluation in our study area [Table 7].

The WHO-recommended 30-cluster sample survey(7) for estimating immunization coverage among infants has been found to be very useful by public health administrators in developing countries, because it is rapid, operationally convenient and cost-effective. Once a very high immunization coverage, say 90%, is attained at the level of a district/city, the public health administrator's concern should shift to the identification of unsatisfactory areas or pockets within this large area that have low coverage to initiate appropriate corrective action since the overall immunization coverage value may mask these low coverage areas.

The WHO 30-cluster survey, undertaken on the large area, cannot detect them, and undertaking a separate survey in every sub-area would be too laborious and expensive. Moreover, the minimum population of any area, to be surveyed for immunization coverage has been recommended to be at least 50,000 to allow for adequate sample size and to be cost-effective.(4) In these circumstances, the adoption of an alternative technique namely, LQAS could prove beneficial.

Similar results have been obtained in studies done elsewhere. As per the study by Murthy *et al.*(8) in Madras City, LQAS technique was useful in identifying ‘unsatisfactory’ pockets in the City, when the overall coverage was satisfactory.

In the study conducted by Valadez *et al.*,(6) LQAS was used to evaluate problems in community health workers' performance. The study demonstrated that the technical quality of vaccination service improved over approximately one year after the introduction of a local supervisory system that used lot quality-assurance sampling.

In the study done by Singh J *et al.*,(3,9,10) it was found that LQAS though not a good substitute for current EPI methodology to evaluate immunization coverage in a large administrative area, it has the potential to monitor health programs on a routine basis in small population sub-units, especially in areas with high and heterogeneously distributed immunization coverage. In addition, LQAS can be applied by local health personnel/ medical officer to know deficient coverage areas in order to focus more on those areas.

## Summary and Conclusion

As per cluster sampling methodology, the percentage of completely immunized children was 84.09%, while partially immunized and unimmunized children were 14.09% and 1.82%, respectively. As per LQAS, overall coverage of completely immunized children was 92.10%. The percentage of partially immunized was 6.58% and unimmunized children accounted for 1.31%. The major source of immunization was urban health center followed by private practitioners, other hospitals and outreach in both the methodologies. The major reasons for dropouts were found to be unaware of need of immunization followed by fear of side reaction.

Immunization coverage evaluated by cluster sampling technique was not statistically significant from the coverage value as obtained by LQAS techniques (*P* > 0.05). Considering the time and resources required, it was found that LQAS is better technique in evaluating the primary immunization coverage in urban area.

The main point of interest that emerges from this study is the practical value of the LQAS technique to the public health administrator in a population which has high overall coverage, where the need is to identify small areas or pockets within the area where the immunisation coverage is deficient. In the present study, there was not much variations found in the coverage amongst the lots. However, LQAS has shown from previous studies that it can be used as a tool to identify the problematic sub-areas so that special action can be initiated which otherwise goes unidentified by an extremely high overall coverage value reported in an area.(5)

## Annexure 1

### Calculation of Sample Size For Cluster Sampling

The sample size is estimated based on the immunization coverage data of Bangalore urban for the year 2004 (data obtained from the district health office, Bangalore urban) and with a precision of 5% and allowable error of 0.05 as follows:

Using the formula

N=Z*Z*p(1−p)/m*m

where *p* is level of immunization = 88%, *m* is allowable error = 0.05, *Z* = 1.96, assumed design effect = 1.25, upper confidence interval = 83.8, lower confidence interval = 92.2 and total sample size estimated was 212.97.

In the study area, 10 clusters were defined based on geographical demarcation.

With the total sample size estimated as 212.97, the sample size for each cluster was 21.297 ≈ 22.

## Annexure 2

**Table:** Calculation of sample size for Lot Quality Assurance Sampling

Lot number	Sub unit	Population	Sample proportion*
1	HMT Layout	15,000	16
	B K Nagar (slum)	3000	3
	Total	18,000	19
2	Thaneerhalli	3020	4
	Akkiyappa Garden	6407	8
	Sanjay Gandhi Nagar	1230	1
	B K Nagar (behind Keshava theatre)	7697	6
	Total	15,334	19
3	B K Nagar	7828	6
	M K Nagar	5772	5
	Brindavan Nagar	9380	8
	Total	22,980	19
4	Pampa Nagar	6000	19
	Total	6000	19

* The sample proportion was done on the basis of the population of the individual areas in each lot
